# Time course changes of anti- and pro-apoptotic proteins in apigenin-induced genotoxicity

**DOI:** 10.1186/1749-8546-8-9

**Published:** 2013-05-04

**Authors:** Fotini Papachristou, Ekaterini Chatzaki, Athanasios Petrou, Ioanna Kougioumtzi, Nikolaos Katsikogiannis, Alexandros Papalambros, Grigorios Tripsianis, Constantinos Simopoulos, Alexandra K Tsaroucha

**Affiliations:** 1 Cell Cultures Unit, Laboratory of Experimental Surgery and Surgical Research, Faculty of Medicine, Democritus University of Thrace, Dragana, Alexandroupolis, Greece; 2 Laboratory of Pharmacology, Faculty of Medicine, Democritus University of Thrace, Dragana, Alexandroupolis, Greece; 3 Postgraduate Program in Hepatobiliary/Pancreatic Surgery, 2nd Department of Surgery, Faculty of Medicine, Democritus University of Thrace, Dragana, Alexandroupolis, Greece; 4 Laboratory of Medical Statistics, Faculty of Medicine, Democritus University of Thrace, Dragana, Alexandroupolis, Greece

## Abstract

**Background:**

Apigenin (4′,5,7-trihydroxyflavone, AP), an active component of many medicinal Chinese herbs, exhibits anticancer properties *in vitro* and *in vivo*. This study aims to investigate the genotoxic, cytostatic, and cytotoxic effects of AP and time course changes in the levels of anti- and pro-apoptotic proteins involved in the DNA damage response in HepG2 cells.

**Methods:**

The genotoxic potential of AP was determined by sister chromatid exchanges (SCEs) and chromosomal aberrations (CAs) analysis. The levels of cytostaticity and cytotoxicity were evaluated by the proliferation rate and mitotic indices, respectively. MTT was used to study cytotoxicity, while the induction of apoptosis and the expression of apoptosis-related proteins were determined by ELISA.

**Results:**

At concentrations greater than 10 μM, AP decreased cell survival in a dose- (48 h: 10 *vs.* 20 μΜ, *P* < 0.001 and 20 *vs.* 50 μΜ, *P* = 0.005; 72 h: 10 *vs.* 20 μΜ, *P* < 0.001 and 20 *vs.* 50 μΜ, *P* = 0.001) and time-dependent manner (20 μΜ: 24 *vs.* 48 h, *P* < 0.001 and 48 *vs.* 72 h, *P* = 0.003; 50 μΜ: 24 *vs.* 48 h, *P* < 0.001 and 48 *vs.* 72 h, *P* < 0.001; 100 μΜ: 24 *vs.* 48 h, *P* < 0.001 and 48 *vs.* 72 h, *P* < 0.001). SCEs rates, cell proliferation, and mitotic divisions were also affected in a dose-dependent manner (*P* < 0.001). There was no change in the frequency of aberrant cells (1 μΜ ΑP: *P* = 0.554; 10 μM AP: *P* = 0.337; 20 μΜ AP: *P* = 0.239). Bcl-2 levels were reduced 3 h after AP administration (*P* = 0.003) and remained reduced throughout the 48 h observation period (6 h, *P* = 0.044; 12 h, *P* = 0.001; 24 h, *P* = 0.042; 48 h, *P* = 0.012). Bax and soluble Fas exhibited a transient upregulation 24 h after AP treatment. The Bax/Bcl-2 ratio was also increased at 12 h and remained increased throughout the 48 h observation period.

**Conclusion:**

AP exhibited dose-dependent genotoxic potential in HepG2 cells. The protein levels of sFas, Bcl-2, and Bax were affected by AP to promote cell survival and cell death, respectively.

## Background

Complementary and alternative medicine has potential to provide new drugs for cancer treatment [1-3]. Plant-derived anticancer agents have already been used in the clinical practice, while many synthetic chemotherapeutics are analogs of natural products [3]. Apigenin (AP) is a bioflavone found in many Chinese medicinal herbs, such as *Wedelia chinensis*, *Ixeris chinensis*, *Apium graveolens* var. *dulce*, *Scutellaria barbata*, *Andrographis paniculata*, *Chrysanthemum morifolium*, and *Ginkgo biloba*[1,4-10]. *In vitro* and *in vivo* studies have demonstrated that AP possesses antioxidant [11,12], anti-inflammatory [13], and anticancer [14-16] properties, inhibiting tumor growth and inducing cell cycle arrest and apoptosis [17-20]. The anticancer properties of AP are associated with its pro-oxidant activity, with concentrations depending upon cell type [21-23]. AP promoted oxidative stress at 15 μΜ in human cervical carcinoma HeLa cells [21], at 50 μΜ in human promyelocytic leukemia HL-60 cells [22], and at 25 μΜ in Chang liver cells [23].

AP induced apoptosis in the hepatic parenchyma [24-29], and exhibited antiproliferative and apoptotic properties in HepG2, Hep3B and PLC/PRF/5 human liver cancer cell lines [25-28]. Its antiproliferative and apoptotic effects might be mediated through a p53-dependent pathway by p53 accumulation, induction of p21 expression, and downregulation of CDK4 expression [25,29]. Generation of reactive oxygen species (ROS) might also play an important role in AP-induced apoptosis by transcriptionally downregulating catalase activity and increasing hydrogen peroxide levels [27,28]. Cell death induction has also been associated with Bax/Bcl-2 ratio changes, cytochrome c release, and Apaf-1 induction, leading to caspase activation and PARP-cleavage in leukemia, prostate carcinoma, lung cancer, and cervical carcinoma cells [19,30-33].

Although the properties of AP against various pro-oxidant and clastogenic agents have been studied [11,34-36], there is little information on the genotoxic potential of this particular flavonoid. AP was highly clastogenic in Chinese hamster V79 cells and induced micronuclei formation in human peripheral lymphocytes in a dose-dependent manner [37,38]. Other reports mentioned that AP could intercalate into both calf thymus DNA and RNA [39,40]. The generation of DNA single-strand (SSBs) and double-strand breaks (DSBs) by DNA-crosslinking agents [41,42], could lead to sister chromatid exchanges (SCEs) or chromosomal aberrations (CAs) [43]. An *in vivo* and *in vitro* study demonstrated that AP can remodel chromatin by inhibiting class I histone deacetylases. This affects regulation, expression, and activation of various DNA damage response genes, which results in cell cycle arrest, and apoptosis. These affected genes include *ATM* and *ATR*, which participate in DSBs repair via homologous recombination [44,45].

The SCEs assay is a sensitive, simple, and rapid method to detect DNA damage and repair at low concentrations of potential genotoxic or anti-genotoxic agents [43,46-49]. SCEs represent a useful tool in monitoring and improving chemotherapeutic strategies *in vitro* and *in vivo*[48,50-54]. The efficacy of potential antitumor agents in inducing SCEs formation *in vitro* and *in vivo* correlates positively with the *in vivo* tumor’s response to these agents [55,56]. CAs analysis is another genotoxic endpoint [43,46]. A high frequency of CAs can lead to cell death, and it has been associated with increased overall cancer risk [43,46,57,58].

AP’s ability to intercalate into DNA, remodel chromatin, and upregulate p53 and p21 proteins [25,39,40,44,59-61] directed us to study the genotoxic potential of this flavonoid in HepG2 cells. We also investigated the proliferation rate index (PRI) and the mitotic index (MI), markers of the cytostatic and cytotoxic properties of chemical and physical agents, respectively [49]. The time course changes in the levels of anti- and pro-apoptotic proteins involved in the DNA damage response were also investigated.

## Methods

### Chemicals

Apigenin (4′,5,7-trihydroxyflavone) was purchased from Calbiochem (San Diego, CA, USA). Bovine serum albumin, Bradford reagent, dimethyl sulfoxide (DMSO), and 3-(4,5-dimethylthiazol-2-yl)-2,5-diphenyl tetrazolium bromide (MTT) were purchased from Sigma (St. Louis, MO, USA). 5-bromodeoxyuridine and bisbenzimide H33258 were purchased from AppliChem (Darmstadt, Germany). High glucose Dulbecco’s modified Eagle’s medium (DMEM), trypsin-EDTA solution, colcemid, fetal bovine serum (FBS), and penicillin/streptomycin solution (10,000:10,000) were purchased from GIBCO (Carlsbad, CA, USA). Cell death detection ΕLISA^Plus^ kit was purchased from Roche (Mannheim, Germany). Human sFas and human sFas ligand ELISA kits were purchased from R&D systems (Minneapolis, MN, USA). Human Bax ELISA kit was purchased from Assay Designs, Inc. (Ann Arbor, MI, USA) and human Bcl-2 ELISA kit was purchased from Bender Medsystems (Vienna, Austria).

### Cell cultures

HepG2 cells were maintained in DMEM supplemented with 10% FBS and 1% penicillin/streptomycin solution, in a 37°C humidified incubator under an atmosphere of 5% CO_2_. On attaining 75–80% confluency the cells were subcultured by trypsinization and then seeded in appropriate cell numbers depending on the type of the experiments. All experiments took place 24 h after seeding.

### Cytotoxicity assay

The cytotoxic potential of AP was evaluated at 24, 48, and 72 h by the MTT method. HepG2 cells were seeded in 96-well plates at a density of 10^4^ cells per well in 100 μL of complete culture medium. Cells were incubated with 0.1, 1, 5, 10, 20, 50, and 100 μΜ of AP or 0.1% DMSO (vehicle control). AP stock solution was prepared in DMSO and diluted in complete culture medium to the desired concentrations (0.1, 1, 5, 10, 20, 50, and 100 μΜ). At each time point, eight replicate cultures for each concentration were studied in three independent experiments. At the end of the specified incubation period (24, 48, and 72 h), the medium was discarded and each well received 200 μL of fresh medium containing 20 μL of MTT solution (5 mg/ml in phosphate buffered saline) for 4 h. MTT crystals were dissolved by adding 100 μL 0.04 M HCL/isopropanol, for fifteen minutes at 37°C. Absorbance was determined at 570 nm by an ExpertPlus microplate reader (ASYS Hitech GmbH, Austria). Absorbance was normalized to vehicle-treated control cultures (equivalent to 100% cell viability).

### SCEs and CAs analysis

For SCEs and CAs determination, 2 × 10^5^ cells were treated with 1, 10, 20, and 50 μΜ of AP and 5 μg/mL of 5-bromodeoxyuridine for 72 h. Colcemid was added to all cultures 24 h before metaphase harvesting. At the end of the incubation period, the cells were scraped and centrifuged (Z300, Hermle Labortechnik GmbH, Germany) at 200 × *g* for 10 min. Pelleted cells were then treated with 5 mL of hypotonic KCl solution (0.075 M), at 37°C for 25 min. Fixative solution (3:1, methanol:acetic acid) was added to the cell suspension and was followed by another centrifugation at 200 × *g* for 10 min. The pellet was washed three times in fixative solution and stored at -20°C until further assayed. All treatments were examined in three independent experiments.

### Fluorescence plus Giemsa

SCEs were visualized by a modified fluorescence plus Giemsa (FPG) technique [62]. Metaphase spreads were incubated in bisbenzimide H33258 solution (0.1 mg/mL) for 20 min at room temperature. A few drops of McIlivaine’s buffer (pH 8; 0.1 M citric acid and 0.2 M disodium phosphate) were applied to each slide and overlaid with a coverslip. After that, the slides were exposed to UV light for 90 min and stained with 7% Giemsa solution in Gurr buffer (pH 6.8).

Since the number of chromosomes in HepG2 cells varies from 50 to 60 (modal number: 55), the SCEs/chromosome rate was estimated. SCEs were evaluated in more than 60 well-spread second-division metaphases for each treatment. CAs were evaluated in 300 first division metaphases for each treatment. The criteria to classify different types of aberrations were in accordance with the recommendations of IPCS guidelines [43]. CAs were classified as chromatid gaps (chtg), chromatid breaks (chtb), chromosome gaps (chrg), chromosome breaks (chrb), ring (r), and dicentric chromosomes (d). Gaps were not included in the determination of total aberrant cells per treatment. Scoring was performed in a blind fashion.

### PRI and MI assessment

For PRI and MI determinations, more than 450 cells and 4,500 nuclei were scored for each treatment, respectively. The proportion of cells in the first, second, third, or subsequent mitotic division was evaluated to determine the PRI. PRI was established according to the following formula:

$$\text{PRI}=\left(\Sigma M_{1}+2*\Sigma M_{2}+3*\Sigma M_{3+}\right)/N$$

where ΣM_1_ is the sum of cells in the first mitotic division, ΣM_2_ in the second and ΣM_3+_ in the third or subsequent mitotic divisions, while N is the total number of cells scored [63]. MI was expressed as the number of cells at metaphase per 1,000 analyzed nuclei (‰).

### Assessment of apoptosis

As an index of apoptosis, cytoplasmic histone-associated DNA fragments were photometrically determined by the Cell Death Detection ΕLISA^Plus^ kit according to the manufacturer’s instructions. Ten thousand cells per well were seeded in 96-well plates and treated with AP (20 μΜ) for 24 h (six replicate cultures). Cells were lysed and centrifuged at 130 × *g*. Supernatants were transferred into a streptavidin-coated microplate and simultaneously incubated with a monoclonal mouse biotinylated anti-histone antibody (clone H11-4) and a monoclonal mouse peroxidase-conjugated anti-DNA antibody (clone MCA-33) at room temperature for 2 h. DNA-histone complex was used as a positive control. Absorbance was determined at 405 nm by an ExpertPlus microplate reader. Background values were subtracted from the measurements.

### Determination of sFas, mFasL, sFasL, Bcl-2, and Bax protein levels

Protein levels of soluble Fas (sFas), membrane-bound Fas-Ligand (mFasL), soluble Fas-Ligand (sFasL), Bcl-2, and Bax were determined by ELISA according to the manufacturers’ instructions. HepG2 cells were treated with 20 μM of AP for 1, 3, 6, 12, 24, and 48 h. Soluble Fas, sFasL, and mFasL protein levels were evaluated in the supernatant or cell lysate of 1 × 10^6^ cells/mL. Bcl-2 and Bax protein levels were determined in 5 × 10^5^ cells/mL and 62,500 cells/mL cell lysates, respectively. Bradford protein assay was used for total protein determination. Protein levels were interpolated from the corresponding standard reference calibration curves. Three independent experiments took place at all time points.

### Statistical analysis

Data were expressed as mean ± standard deviation (SD). SCEs and CAs values were logarithmically transformed before further analysis because data were not normally distributed. Statistical analysis was performed by Student’s *t*-test for individual comparisons between control and apigenin-treated cultures. Multiple comparisons among various AP treatments were carried out by one-way ANOVA followed by Bonferroni’s *post hoc* test. Linear regression analysis was used to determine dose–response relationships. Pearson’s correlation coefficient was also determined. All statistical analyses were performed by SPPS version 16 (IBM, USA). All tests were two-tailed and *P* values less than 0.05 were considered statistically significant. For Student’s *t*-test, significance levels were adjusted to 0.01 to reduce the overall Type I error.

## Results and discussion

**Figure 1:**
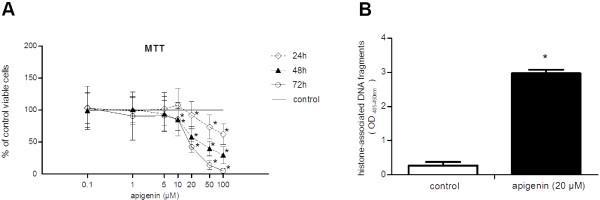
**The effect of apigenin on cell viability and induction of apoptosis. A**. The cytotoxic and antiproliferative effect of apigenin in HepG2 cells. **B**. The apoptotic potential of apigenin (20 μΜ) after 24 h of treatment. The star (*) indicates statistical significance compared with control cultures.

AP concentrations ranging from 10 to 50 μΜ at 48 and 72 h showed a dose–response relationship of cell survival (48 h: 10 *vs.* 20 μΜ, *P* < 0.001 and 20 *vs.* 50 μΜ, *P* = 0.005; 72 h: 10 *vs.* 20 μΜ, *P* < 0.001 and 20 *vs.* 50 μΜ, *P* = 0.001), while AP concentrations ranging from 20–100 μΜ showed a time-dependent decrease (20 μΜ: 24 *vs.* 48 h, *P* < 0.001 and 48 *vs.* 72 h, *P* = 0.003; 50 μΜ: 24 *vs.* 48 h, *P* < 0.001 and 48 *vs.* 72 h, *P* < 0.001; 100 μΜ: 24 *vs.* 48 h, *P* < 0.001 and 48 *vs.* 72 h, *P* < 0.001) (48 h: IC_50_ = 34.58 μΜ; 72 h: IC_50_ = 18.80 μΜ) (Figure 1A). Similar results were reported by Chiang *et al.*[25] and Choi *et al.*[27], who studied the antiproliferative effect of AP in HepG2 cells. In agreement with Khan *et al.*[26], AP induced apoptosis at 24 h, eliciting an 11-fold increase in cytoplasmic histone-associated DNA fragments (*P* < 0.001) (Figure 1B).

AP (100 μΜ) induced DNA damage in Chinese hamster V79 cells and increased the frequency of micronuclei and CAs (concentrations higher than 37 μM) in human peripheral lymphocytes [34,36-38,64,65]. In our study, we observed no effect on the frequency of aberrant cells (Table 1) but we noticed an increase in the frequency of SCEs at much lower concentrations (10 and 20 μΜ). However, 50 μΜ of AP was highly cytotoxic, making SCEs evaluation impossible (Table 2). This could be attributed to the high sensitivity of the SCEs method for detecting DNA damage and repair at doses that have little or no effect on CAs frequency [46-49,66]. Moreover, the independent mechanisms leading to SCEs and CAs formation [46,67-69] and the use of different cell systems could account for the differences. HepG2 cells have a polymorphic genetic profile with a variety of structural and numerical chromosomal abnormalities [70-72]. Sixty-seven breakpoints were identified in liver cancer cell lines including HepG2 cells [72]. Zimonjic *et al.*[71] performed comparative genomic hybridization analysis in 18 liver cancer cell lines and reported that regions exhibiting gain or loss, ranged from whole chromosome arms to a medium band of a 400-band ideogram. These previous findings could justify the high frequency of aberrant cells found in the control groups in the present study.

**Table 1:** The effect of apigenin on the frequency of chromosomal aberrations

	**No. cells scored**	**% of aberrant cells (± SD)**	**Chromosomal aberrations/cell**					
			**chtg**	**chtb/f**	**chrg**	**chrb/f**	**r**	**d**
Untreated control	300	55.8 ± 14.27	0.16 ± 0.03	0.51 ± 0.15	0.10 ± 0.07	0.20 ± 0.15	0.035 ± 0.03	0.010 ± 0.01
Vehicle control	300	60.5 ± 5.05	0.15 ± 0.09	0.63 ± 0.19	0.05 ± 0.03	0.32 ± 0.06	0.003 ± 0.01	0.003 ± 0.01
AP (1 μM)	303	56.2 ± 12.57	0.08 ± 0.01	0.49 ± 0.09	0.05 ± 0.03	0.27 ± 0.18	0.013 ± 0.02	0.003 ± 0.01
ΑP (10 μΜ)	300	68.2 ± 13.88	0.13 ± 0.07	0.63 ± 0.12	0.06 ± 0.02	0.33 ± 0.16	0.022 ± 0.03	0.000 ± 0.00
AP (20 μΜ)	300	66.0 ± 6.60	0.16 ± 0.06	0.56 ± 0.05	0.07 ± 0.03	0.41 ± 0.03	0.024 ± 0.02	0.010 ± 0.01

chtg: chromatid gap; chtb: chromatid break; f: fragment; chrg: chromosome gap; chrb: chromosome break; r: ring; d: dicentric.

**Table 2:** The genotoxic, cytostatic, and cytotoxic potential of apigenin

**Agents**	**SCEs/chromosome ± SD (range of values)**	**PRI**	**MI (‰)**
Untreated control	0.17 ± 0.08 (0.04-0.42)	2.64 ± 0.03	76.3 ± 9.50
Vehicle control	0.15 ± 0.08 (0.03-0.33)	2.65 ± 0.06	87.3 ± 20.20
AP (1 μM)	0.15 ± 0.09 (0.04-0.42)	2.61 ± 0.10	63.0 ± 19.52
ΑP (10 μΜ)	0.22 ± 0.14*^,a ^(0.03-0.69)	2.52 ± 0.13	57.0 ± 22.84
AP (20 μΜ)	0.26 ± 0.12*^,b,c ^(0.07-0.64)	2.09 ± 0.12*^,d^	32.7 ± 7.96*
ΑP (50 μΜ)	ND	ND	9.3 ± 7.57*^,e^

*P ≤ 0.01 *vs. *vehicle control; ^a^*P* < 0.01 *vs. *1 μM; ^b^*P* < 0.001 *vs. *1 μM; ^c^*P* < 0.05 *vs. *10 μΜ; ^d^*P* ≤ 0.001 *vs. *1 and 10 μΜ; ^e^*P* < 0.01 *vs. *1, 10 and 20 μM. ND: values could not be determined.

Linear regression analysis revealed a dose–response relationship between AP and SCEs frequencies, cell proliferation, and mitotic divisions (R = 0.798, R^2^ = 0.636, *P* < 0.001; R = -0.883, R^2^ = 0.781, *P* < 0.001; R = -0.820, R^2^ = 0.672, *P* < 0.001, respectively). AP concentration was positively correlated with the first and second mitotic division metaphases (R = 0.837, R^2^ = 0.700, *P* < 0.001; R = 0.768, R^2^ = 0.589, *P* < 0.001, respectively), while the third and subsequent mitotic division metaphases were negatively correlated (R = -0.867, R^2^ = 0.751, *P* < 0.001) with AP (Table 3). The flavonoid’s genotoxic potential was correlated with increased cytostaticity (SCEs *vs.* PRI: R = -0.582, R^2^ = 0.339, *P* = 0.018) and cytotoxicity (SCEs *vs.* MI: R = -0.573, R^2^ = 0.329, *P* = 0.032).

**Table 3:** The effect of apigenin on cell cycle kinetics

**Agents**	**Mean (± SD) number of cells in the 1**^**st**^**, 2**^**nd**^**, 3**^**rd**^**, and subsequent mitotic divisions**		
	**1**^**st**^	**2**^**nd**^	**3**^**rd+**^
Untreated control	4.3 ± 1.15	39.0 ± 9.54	89.7 ± 20.55
Vehicle control	5.0 ± 2.65	42.0 ± 10.44	103.0 ± 9.17
AP (1 μM)	4.0 ± 2.65	50.0 ± 9.54	97.3 ± 14.05
ΑP (10 μΜ)	7.4 ± 3.65	57.2 ± 16.57	85.2 ± 17.34
AP (20 μΜ)	20.2 ± 5.40*^,a,b^	89.4 ± 20.38*^,c^	34.2 ± 16.45*^,a,d^

*P ≤ 0.01 *vs. *vehicle control; ^a^*P* < 0.01 *vs. *1 μM; ^b^*P* < 0.05 *vs. *10 μΜ; ^c^*P* < 0.05 *vs. *1 and 10 μΜ; ^d^*P* < 0.01 *vs. *10 μΜ.

**Figure 2:**
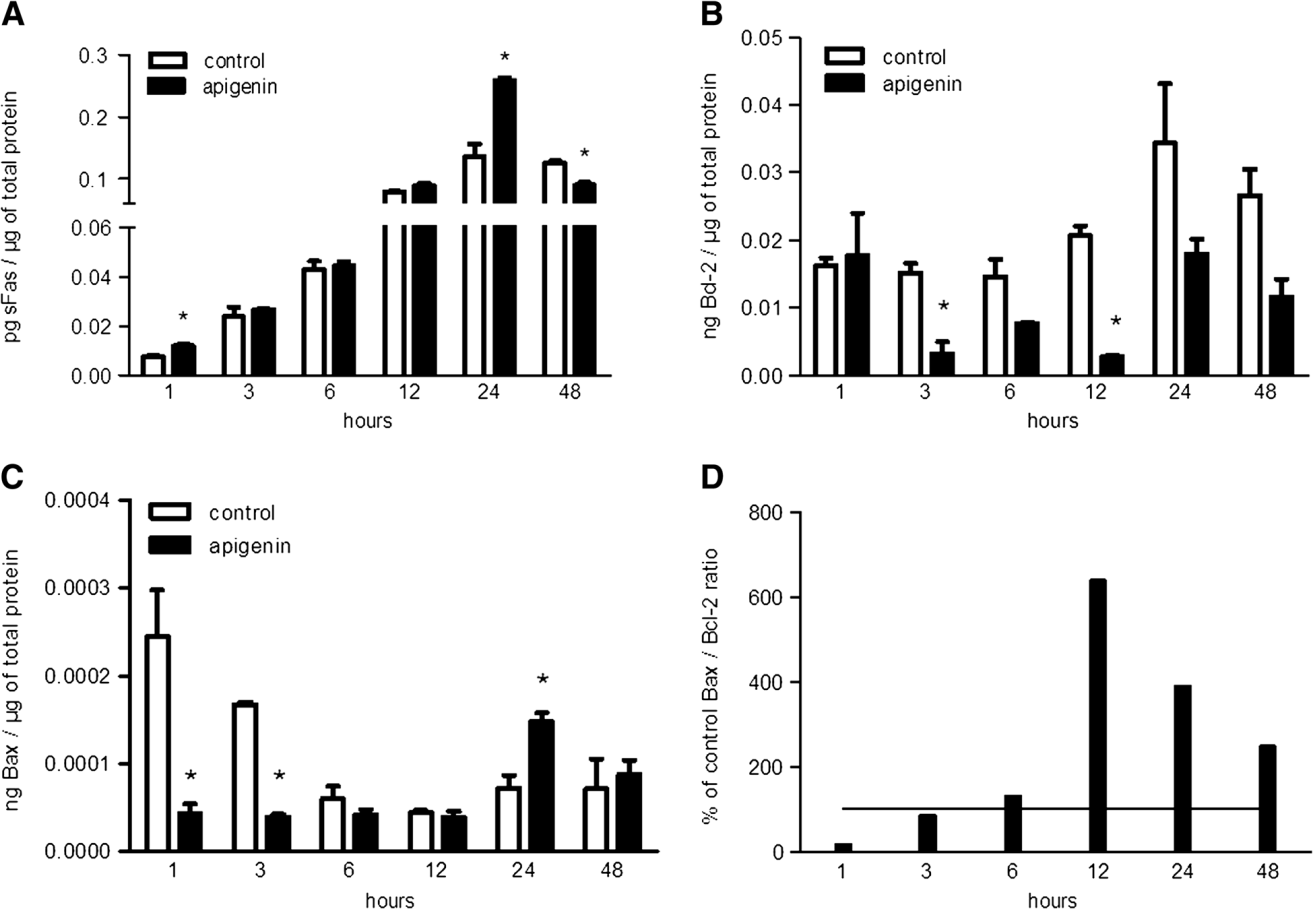
**The effect of apigenin on apoptosis-related proteins. A**. The effect of apigenin (20 μΜ) treatment on sFas protein levels. Control cultures: 6 *vs. *12 h, *P* = 0.005; 12 *vs. *24 h, *P* = 0.001. Apigenin treatment: 3 *vs. *6 h, *P* = 0.021; 6 *vs. *12 h, *P* = 0.001; 12 *vs. *24 h, *P* = 0.001; 24 *vs.* 48 h, *P* = 0.001. **B**. The effect of apigenin (20 μΜ) treatment on Bcl-2 protein levels. **C**. The effect of apigenin (20 μΜ) treatment on Bax protein levels. **D**. The effect of apigenin (20 μΜ) treatment on Bax/Bcl-2 ratio. According to linear regression analysis: Time intervals 1–12 h, R = 0.960, R^2^ = 0.921, *P* = 0.040; Time intervals 12–48 h, R = -0.957, R^2^ = 0.915, *P* = 0.188. The star (*) indicates statistical significance compared with the respective control cultures.

DSBs are repaired by homologous recombination, in which SCEs play an important role [73-75]. Iijima *et al.*[76] reported that NBS1, a protein involved in cellular responses to DSBs [77], regulated Bax activation in DNA damage-induced apoptosis. Furthermore, pro-apoptotic *BAX* could comprise a p53 downstream target gene through the direct binding of p53 to cofactors ASPP1 and ASPP2 [78,79]. Bcl-2 could protect cells against cell death induced by ionizing radiation, alkylating agents, and various chemotherapeutic drugs [80-83]. Formation of DSBs by severe DNA damage triggered Bcl-2 decline and activated caspase-9 and caspase-3 [84]. In our study, AP treatment affected both Bcl-2 and Bax protein levels. Bcl-2 expression was downregulated at 3–48 h (Figure 2B). Bax levels were significantly lower in apigenin-treated cells at 1 and 3 h (*P* = 0.003 and *P* < 0.001, respectively) (Figure 2C). At 24 h, Bax expression was significantly upregulated compared with the corresponding vehicle-treated cultures (*P* = 0.005) and the respective 12 h of treatment (*P* = 0.001). Bax’s upregulation was transient and significantly reduced to the corresponding control cultures levels after 48 h. The Bax/Bcl-2 ratio, which is indicative of the mitochondrial induced apoptotic potential, exhibited a noticeable increase from 12–48 h of treatment (Figure 2D). Similar changes in the Bax/Bcl-2 ratio were observed in human lung A549 cancer cells and human prostate carcinoma DU145 cells [32,33].

Genotoxic agents could lead to cell death through the Fas/FasL mediated apoptotic pathway [85,86]. In the present study, mFasL and sFasL were undetectable at all time points in all cultures. Nevertheless, sFas increased in a time-dependent manner in untreated and treated cultures (Control cultures: 6 *vs.* 12 h, *P* = 0.005; 12 *vs.* 24 h, *P* = 0.001. Apigenin treatment: 3 *vs.* 6h, *P* = 0.021; 6 *vs.* 12 h, *P* = 0.001; 12 *vs.* 24 h, *P* = 0.001; 24 *vs.* 48 h, *P* = 0.001) (Figure 2A). AP increased sFas levels at 1 and 24 h, compared with the corresponding control cultures (*P* = 0.002 and *P* = 0.001, respectively). Upregulation of sFas at 24 h, was transient and significantly reduced at 48 h, compared with the respective 24 h treatment (*P* = 0.001) and the corresponding control cultures (*P* = 0.001). There are no previous reports on the effect of AP on alternatively spliced *FAS*. Fas-mediated signaling is not limited to inducing cell death, and its expression in various cell types does not always correlate with susceptibility to the Fas-mediated apoptotic pathway [86,87]. Alternatively, spliced *FAS* variants, which encode soluble forms of the receptor, could inhibit apoptosis [88-90]. Because of the sharp Bcl-2 downregulation at 12 h, sFas upregulation might represent a rescuing mechanism as a means to prevail over cell death signals. Filippov *et al.*[91] reported that cells in response to exogenous stress, such as the effect of a genotoxic agent, regulated the expression of specific splicing factors, altering the splicing profile of target genes such as *CD44* and *FAS*. Since ROS induce alternative splicing, it is possible that free oxygen radical generation by AP could justify the induction of sFas expression [27,92,93]. AP’s intercalation into DNA/RNA might have also contributed to these changes in the expression of *FAS*.

## Conclusion

AP exhibited dose-dependent genotoxic potential that led to changes in sFas, Bcl-2, and Bax protein levels in HepG2 cells.

## Abbreviations

AP: Apigenin; CAs: Chromosomal aberrations; chrb: Chromosome break chrg, chromosome gap; chtb: Chromatid break; chtg: Chromatid gap; d: Dicentric chromosome; DMEM: Dulbecco’s modified Eagle’s medium; DMSO: Dimethyl sulfoxide; DSBs: Double-strand breaks; f: Fragment; FBS: Fetal bovine serum; mFas: Membrane-bound Fas; mFasL: Membrane-bound Fas-Ligand; MI: Mitotic index; MTT: 3-(4,5-dimethylthiazol-2-yl)-2,5-diphenyl tetrazolium bromide; PRI: Proliferation rate index; r: Ring chromosome; ROS: Reactive oxygen species; SCEs: Sister chromatid exchanges; SD: Standard deviation; sFas: Soluble Fas; sFasL: Soluble Fas-Ligand; SSBs: Single-strand breaks.

## Competing interests

The authors declare that they have no competing interests.

## Authors’ contributions

CS, EC and AKT designed and supervised the study. FP wrote the manuscript and conducted the experiments. AtP, IK, NK, and AlP assisted experimentally and drafted parts of the manuscript. GT and FP performed the statistical analysis. All authors read and approved the final version of the manuscript.
